# The Effectiveness of Measuring Thiopurine Metabolites in the Treatment of Autoimmune Hepatitis Patients

**DOI:** 10.5152/tjg.2024.22838

**Published:** 2024-03-01

**Authors:** Woroud Yassin, Roni Nasser, Ella Veitsman, Tarek Saadi

**Affiliations:** 1 Israel Institute of Technology, The Ruth and Bruce Rappaport Faculty of Medicine, Haifa, Israel; 2 Department of Liver, Rambam Health Care Campus, Haifa, Israel; 3 Department of Gastroenterology, Rambam Health Care Campus, Haifa, Israel

**Keywords:** Autoimmune hepatitis, thiopurine drugs, shunting

## Abstract

**Background/Aims:**

The thiopurine drugs—azathioprine and mercaptopurine—are purine antimetabolites used for the treatment of autoimmune hepatitis. These drugs undergo metabolism through genetically determined pathways, which influences their effectiveness and toxicity. There is scarce information regarding the clinical effects of measuring drug metabolites in these patients. The goal of the study is to test the clinical significance of measuring thiopurine metabolites in patients unsuccessfully treated with thiopurines.

**Materials and Methods:**

Clinical and laboratory data collected for patients who were treated for autoimmune hepatitis between 2015 and 2018, and did not achieve full remission under thiopurine therapy and had thiopurine metabolite levels measured due to lack of response and suspicious side effects were chosen. We compared clinical and laboratory data before and after the therapy change.

**Results:**

The study included 25 tests of thiopurine metabolites in 21 patients. Six tests had therapeutic levels. Three tests showed high levels leading to lowering the drug dose. In 11 cases, levels of 6-thioguanine nucleotide were low; the dose was not changed in 3 of these, and the dose was increased in the remaining 8. Shunting was observed in 5 cases, 2 of which were mild and the dose was not changed. In the remaining 3, the dose was decreased, and allopurinol was added. Significant improvements in liver enzymes were observed following dose adjustments.

**Conclusion:**

We showed that, in cases of suboptimal response to thiopurine treatment, measuring thiopurine metabolites had an important role in optimizing therapy. In most patients, changing the dose led to a significant improvement with no need to switch to second-line therapies.

**Main Points**

- Thiopurines are the main drugs for maintenance in autoimmune hepatitis.
- These drugs undergo metabolism through genetically determined pathways, which influences their effectiveness and toxicity.
- In cases of suboptimal response to thiopurine treatment, measuring thiopurine metabolites had an important role in optimizing therapy which can lead to significant improvement.

## Introduction

Autoimmune hepatitis is a chronic inflammatory disease affecting the liver, characterized by a relapsing and remitting course. If untreated, it could lead to cirrhosis, liver failure, and even death. It responds well to immunosuppressive therapy, mainly steroids as monotherapy or as a combination with thiopurines.^1-3^

Thiopurines—azathioprine (AZA) and mercaptopurine (MP)—are drugs commonly used in remission maintenance.^4^ These drugs can have serious side effects which led to drug discontinuation in up to 15% of treated individuals.^5^ Broadly, side effects fall into 1 of 2 groups: dose related or idiosyncratic. Rash, fever, and joint pain are the main nondose-related side effects; however, hepatitis and pancreatitis could also appear regardless of drug dose. Drug-induced liver injury (DILI) falls on a spectrum, ranging from asymptomatic elevation of liver enzymes, necrosis of liver cells and cholestasis to mixed injury. Moreover, injury to the vascular endothelium of the liver was observed, presenting as sinus dilatation, peliosis, nodular regenerative hyperplasia, and obstruction of liver veins. The main and the most serious dose-related side effect is bone marrow suppression.^5,6^

**Figure 1.**
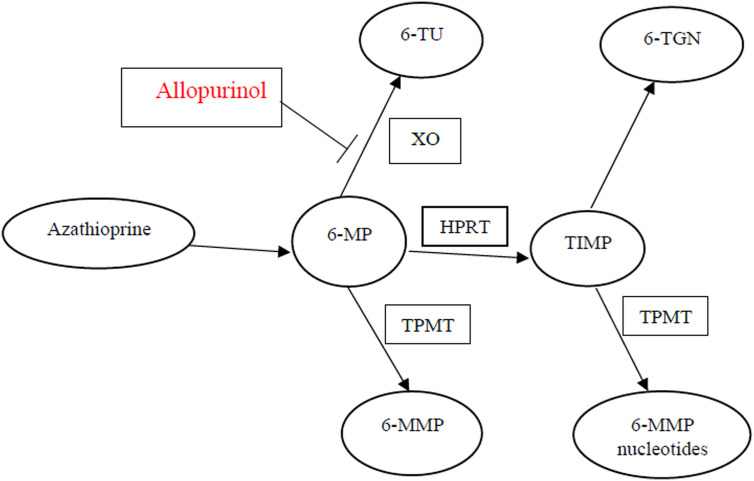
Metabolism of azathiopurine, cleaved to form 6-MP after an oral dose. Further metabolism occurs through competing pathways by 3 different enzymes: XO, TPMT, and HPRT. TPMT, thiopurine methyltransferase. XO, Xanthine oxidoreductase; HPRT, hypoxanthine phosphoribosyltransferase.

Thiopurine metabolism (Figure 1) depends on genetically determined enzymatic pathways.^7-10^ After an oral dose, 88% of the drug is nonenzymatically converted to 6-MP, which is metabolized either to 6-methylmercaptopurine (6-MMP) which has no therapeutic effects or to the active metabolite 6-thioguanine nucleotides (6-TGNs) which are responsible for both the cytotoxic and immunosuppressive actions.^10,11^

Thiopurine methyltransferase (TPMT) is the enzyme responsible for the production of 6-MMP.^12^ Its activity is reliant on a genetic polymorphism, and it is estimated that 11% of the population shows reduced activity due to heterozygotic mutation, while 0.3% shows negligible activity linked to homozygotic mutation.^13,14^ Phenotypes with reduced TPMT activity have a higher risk of developing bone marrow suppression due to higher concentrations of 6-TGN, secondary to the reduced enzymatic activity.^9,11,15^ Conversely, phenotypes with increased TPMT activity tend to display low 6-TGN concentrations high 6-MMP concentrations, a phenomenon known as shunting; these patients are less responsive to therapy.^16^

A number of studies have demonstrated that 6-MMP concentrations higher than 5700 pmol/8 × 10^8^ red blood cells (RBCs) are associated with liver toxicity and, in extremely high concentrations, with bone marrow toxicity.^16-19^

Allopurinol is an Xanthine oxidoreductase (XO) inhibitor and is used to treat gout and other diseases. It has been suggested that allopurinol metabolites could inhibit 6-MP to 6-thioric acid conversion. Accordingly, adding allopurinol to thiopurine treatment would channel the metabolism toward 6-TGN instead of 6-MMP.^20-22^

Studies in IBD patients have suggested that 6-TGN levels between 250 and 450 pmol/8 × 10^8^ RBCs are therapeutic^19,23^ and that lower or higher levels are respectively associated with reduced efficacy or increased bone marrow suppression risk.

There is limited data regarding monitoring thiopurine metabolites in patients suffering from autoimmune hepatitis. According to the European Association for the Study of the Liver (EASL) guidelines, it is possible to consider monitoring for thiopurine metabolite levels in cases of suboptimal response to therapy.^24^ We aimed to investigate the clinical impact of monitoring thiopurine metabolite levels and the therapeutic interventions made after these determinations, on patients suffering from autoimmune hepatitis with suboptimal response to therapy.

## Materials and Methods

### Study Design

Patients 18 years old or above suffering from autoimmune hepatitis and treated between the years 2015 and 2018 were investigated, using The Rambam Health Care Campus (RHCC) Hospital’s integrated electronic medical records system. 

The Rambam Health Care Campus (RHCC) Hospital Institutional Review Board (Approval No: RMB0415-17, Date: 14/12/2017) approved the study according to Helsinki. There are about 300 patients with autoimmune diseases (including those with overlap syndromes) in our clinic registry. Patients younger than 18 years and those with other liver diseases were excluded. Due to the retrospective type of the study, the need for informed consent was waived. Patients who did not have full remission under thiopurine therapy and had thiopurine metabolite levels measured due to lack of response, suspicious side effects (leukopenia, hepatitis) were included. Other etiologies for abnormal liver tests were excluded with full revision on patients recording including new drugs, blood tests, and imaging. The thiopurine metabolite levels were measured by high-performance liquid chromatography method. A high-performance liquid chromatography method capable of measuring thiopurine mono-, di-, and triphosphates separately in RBCs were isolated from whole blood using centrifugation. Proteins were precipitated using dichloromethane and methanol. The TGNs were derivatized using potassium permanganate before analysis. Analytes were separated by ion-pairing liquid chromatography using tetrabutylammonium ions and detected using ultraviolet absorption and fluorescence.

Demographic, clinical, and laboratory data were collected, in addition to any regimen change made after the measurement of drug levels. We compared clinical and laboratory data before and after the therapy change. 

In patients with low metabolite levels, patients were encouraged to take the drugs. Another cause could be that the drug dose is insufficient, and the proper action in such a case would be to raise the dose. Shunting could also be a correctable cause for lack of efficacy; in such a case, lowering the dose and adding allopurinol would lead to the desired improvement. In patients with toxic levels, the dose was decreased.

The following data were retrieved from the electronic medical records: age, gender, weight; laboratory values including hemoglobin (Hb), white blood cell (WBC) count, platelets (PLT), serum glucose, serum creatinine, blood urea nitrogen, albumin, aspartate transaminase (AST), alanine transaminase (ALT), gamma-glutamyl transferase (GGT), alkaline phosphatase (ALKP), bilirubin, and immunoglobulin G (IGG) levels.

### Statistical Analysis

Descriptive statistics in terms of mean ± SD, median, and percentage were performed on the whole parameters in the study. Normal distribution for the quantitative parameters was examined by Shapiro–Wilk test. Since we had a small sample size, determining the distribution of the variable parameters was important for choosing an appropriate statistical method. So a Shapiro–Wilk test was performed and showed that the distribution of the parameters departed significantly from normality. Based on this outcome, a nonparametric test was used, and the median with the interquartile range was used to summarize the variables. Lab tests before and after therapy modification were tested by paired tests. Statistical Package for the Social Sciences version 25.0 (IBM Inc., Armonk, NY, USA) was used for statistical analysis. *P* < .05 was considered as significant. 

## Results

Twenty-one patients who underwent a total of 25 blood tests for thiopurine metabolites were included in the study. The test was repeated in cases of continuous signs of liver injury after therapy modification or in cases of borderline results. The tests were repeated in 4 patients because of ongoing signs of liver injury after therapy modification in 3 patients and borderline results in 1 patient.

Table 1 sums patients’ demographic and clinical characteristics at presentation. A total of 64% of the participants were female and 36% were male. The mean age of the participants was 51.2 years, and the average weight was 78.7 kg. None of the patients have cirrhosis. The participants’ liver function tests were, on average, elevated, and their WBC count was in the upper range of normal. Red blood cells, PLT, and thyroid functions were normal.

**Figure 2.**
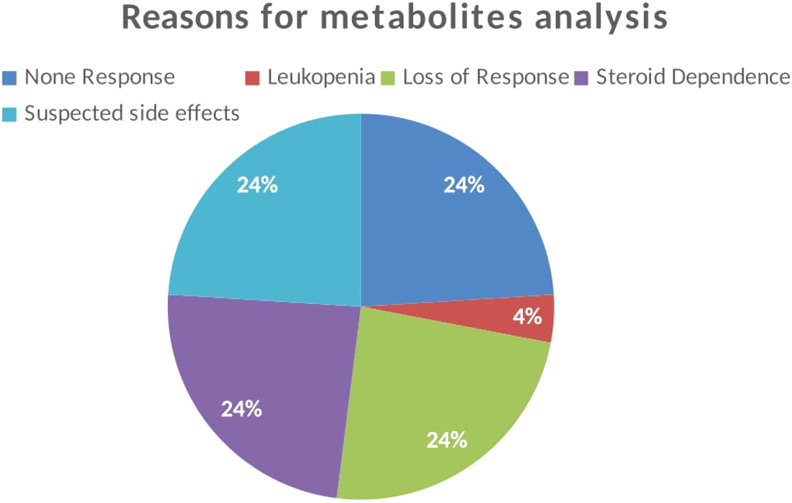
The reasons for testing for thiopurine levels in the study participants.

The reasons for testing thiopurine metabolite levels were lack of response, decrease in response after continuous therapy, steroid dependence, and because of suspected side effects such as leukopenia and hepatitis as mentioned in Figure 2. A single patient had the test done due to the serious complication of leukopenia.

Table 2 contains 6-TGN and 6-MMP levels and their ratios for the various participants, in addition to any changes performed to the therapeutic regimen, including the addition of allopurinol. Utilizing this information and follow-up notes in the patient’s file, it was decided if the findings were consistent with low drug dose, toxic dose, or shunting for each case. Eleven cases were consistent with the clinical findings in low dose, that is, low 6-TGN and 6-MMP that were not elevated. Most of these had their drug dose increased if there were no contraindications. There were 5 cases of shunting, 3 of which had their doses decreased. The other 2 had no changes made to their therapy. In these cases, 6-MMP levels were in the normal range, but the ratio was elevated.

Four patients had allopurinol added to their regimen at a dose of 100 mg daily. These included the 3 cases of shunting in whom the thiopurine dose was decreased and 1 patient who was classified as having a high dose, although he was treated with a relatively low initial dose of 50 mg of AZA. None of the patients required switching to any second-line therapy, such as tacrolimus or cyclosporin.

**Figure 3.**
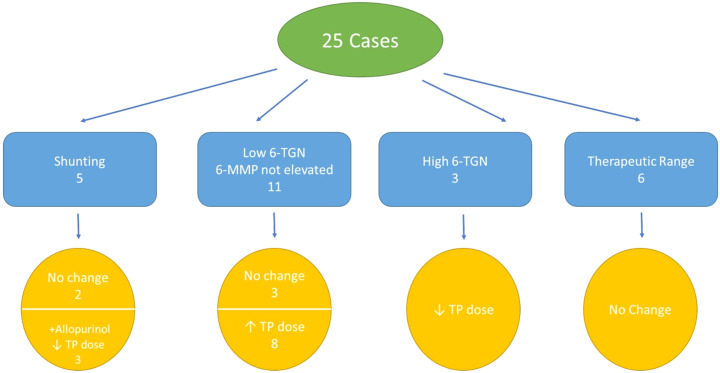
Therapeutic changes made to study patients after measuring their metabolite levels.

Seventeen patients out of 21 (81%) have normalized their liver test after modification was done. Figure 3 reports the different therapeutic interventions performed on the patients in the study.

A statistically significant improvement was observed in all the liver enzymes following therapy modification. Alanine transaminase dropped to 32.96 on average (*P *< .001), AST to 30.36 (*P *< .001], ALPK to 104.56 (*P *= .001), and GGT to 78.49 (*P *= .011) as mentioned in Table 1.

Statistically nonsignificant improvements were observed in bilirubin, IGG, and WBC. No adverse effects were documented following therapy modification.

## Discussion

Thiopurines remain first-line and the most effective therapy for autoimmune hepatitis.^1-3^ However, at times, the therapy fails to achieve clinical improvements or is hampered by side effects or intolerance, necessitating a switch to less effective and more troublesome second-line agents. Monitoring blood levels of thiopurine metabolites and adjusting the dose accordingly is a common and accepted practice in patients suffering from IBD. A study performed by Dhaliwal et al^18^ demonstrated that 6-TGN levels above 220 pmol/8 × 10^8^ RBC are predictive of better therapy outcomes in patients suffering from autoimmune hepatitis. The 6-TGN levels between 250 and 450 pmol/8 × 10^8^ RBC are considered therapeutic in these patients^19,23^ and lower or higher levels are, respectively, associated with reduced efficacy or increased bone marrow suppression risks.

As mentioned earlier, according to EASL guidelines, it is possible to consider monitoring for thiopurine metabolite levels in cases of suboptimal response to therapy.^24^ American Association for the Study of Liver Diseases (AASLD) does not provide any guidelines in this regard.^25^

In this study, we aimed to investigate the clinical impact of monitoring thiopurine metabolite levels and the therapeutic interventions made after these determinations on patients suffering from autoimmune hepatitis with suboptimal response to therapy. In some cases, this lack of efficacy is due to low adherence, toxic dose, or low dose. In contrast to patients suffering from IBD, thiopurine metabolite levels are not routinely measured in autoimmune hepatitis patients, in order to determine the mechanism of their lack of efficacy.^19,23^ In this study, as stated earlier, these levels were measured in autoimmune hepatitis patients who failed to respond to therapy.

Only in 6 of the 25 cases in this study were the metabolite levels in the normal range, meaning that the rest had a possibly correctable cause for the therapy’s lack of efficacy. Eleven patients had low 6-TGN levels, 8 of whom had their dose consequently increased. Five cases had shunting, 3 of whom had their doses decreased and allopurinol added to their regimen.

There was no need to switch to second-line therapy in any of the patients, which most likely would have been the case had metabolite levels not been measured. This fact reflects the importance of thiopurine metabolite levels measurement in cases of low or no efficacy, and its role in identifying correctable causes for therapy failure, such as low tolerance, which would present as negligible metabolite levels. In such cases, the proper action would be to encourage the patient to take the drugs. Another cause could be that the drug dose is insufficient, and the proper action in such a case would be to raise the dose. Shunting could also be a correctable cause for lack of efficacy; in such a case, lowering the dose and adding allopurinol would lead to the desired improvement.

This study demonstrated that lack of response to thiopurine therapy is due to correctable causes in most cases. Similar to IBD patients, measuring thiopurine metabolite levels in autoimmune hepatitis patients led to a better understanding of the cause behind the lack of response to therapy and, consequently, to appropriate therapeutic interventions that, in most cases, led to significant improvement.

This study has several shortcomings. First, it is a retrospective study, performed on a relatively small number of patients. Second, interpreting the metabolite levels and the therapy modifications were performed according to the clinician’s judgment, or guidelines from other diseases, such as IBD. This is a result of the fact that measuring thiopurine metabolite levels has yet to enter mainstream practice in autoimmune hepatitis and, so far, no proper guidelines have been issued. Third, the study does not have a control group to determine the level of metabolites in patients with optimal responses. So we were not able to assess the specificity of these tests. Fourth, due to the retrospective nature of the study, drug compliance could not be determined.

In conclusion, our study showed that measuring thiopurine metabolite levels is important for follow-up and therapy optimization in autoimmune hepatitis patients. Further studies are needed, on a larger scale, preferably utilizing a prospective approach and relying on uniform decision-making algorithms, to properly assess the significance of measuring thiopurine metabolite levels in autoimmune hepatitis.

## Figures and Tables

**Table 1.** Patient Characteristics and Lab Results at Presentation and After Drug Modification

	Normal Values	Mean	Minimum	Maximum	Median Pre (25%-75%)	Median Post (25%-75%)	*P*
Age, mean (year)	3	51.2	18	76.1	3	3	3
ALT, mean (U/L)	0-55	78.4	10	354	49 (36-95.5)	27 (18-49)	<.0001
AST, mean (U/L)	5-34	75.1	18	364	46 (26.5-85)	31 (20.5-39)	<.0001
ALKP, mean (U/L)	40-150	152.8	50	595	97 (72-187)	80 (64.5-146)	=.001
GGT, mean (U/L)	12-64	163.8	13	565	110.5 (33.5-307.5)	32 (17-70.5)	.011
Bilirubin, mean (mg/dL)	0.2-1.2	0.9	0.3	4.3	0.60 (0.49-0.83)	0.55 (0.40-0.68)	.086
IgG, mean (U/L)	680-1560	1373.9	0.7	3437	1336 (1016-1673)	1296 (907-1524)	.21
WBC, mean (1/µL)	4-10.8	9187.6	1440	90 600	5500 (4550-7400)	5400 (4190-6550)	.16
Hb, mean (g/dL)	11.5-15	13.1	10	17.2	12.8 (12-14.25)	12.6 (11.8-14.0)	.32
PLT, mean (1/µL)	130-400	241 800	71 000	430 000	239 000 (191 000-281 000)	220 000 (202 000-287 500)	.82

ALKP, alkaline phosphatase; ALT, alanine transaminase; AST, aspartate transaminase; GGT, gamma-glutamyl transferase; IgG, Immunoglobulin G; Hb, hemoglobin; PLT, platelets; WBC, white blood cells.

**Table 2.** Metabolite Levels and Modified Therapy

Patient Number	Fibrosis Status	Test No.	Thiopurine Type	Original Therapy					Ratio	Lab Analysis	Modified Therapy			
				Duration of Treatment (Months)	Thiopurine Dose (mg)	Steroid Dose (mg)	6-TGN Level (pmol/q)	6-MMP Level (pmol/q)			Thiopurine Type	Thiopurine Dose (mg)	Steroid Dose (mg)	Allopurinol Dose (mg)
1	F3	1	Azathioprine	36	100	10	174	4256	24.4	Shunting	Azathioprine	50	10	100
3	F3	2	Azathioprine	48	50	20	511	568	1.11	Toxic dose	Azathioprine	25/50*	7.5	100
2	F1	3	Azathioprine	12	100	5	131	955	7.29	UD	Azathioprine	150	5	0
3	F1	4	Azathioprine	18	150	5	200	1870	9.36	UD	Azathioprine	150	0	0
3	F2	5	Azathioprine	30	75	7.5	86	120	1.4	UD	Azathioprine	100	0	0
4	F1	6	6-MP	12	75	5	141	>10000	3	Shunting	6-MP	25/50*	0	100
5	F2	7	Azathioprine	6	75	5	209	77	0.36	UD	Azathioprine	100	0	0
3	F2	8	Azathioprine	12	100	0	89	581	6.53	UD	Azathioprine	100	0	0
6	F2	9	Azathioprine	12	150	20	162	>10000	3	Shunting	Azathioprine	50	5	100
7	F1	10	Azathioprine	24	75	0	243	616	2.53	Response	Azathioprine	75	0	0
8	F1	11	Azathioprine	12	50	10	239	805	2.79	Response	Azathioprine	150	5	0
9	F4	12	Azathioprine	12	50	20	205	222	1.08	UD	None	0	10	0
10	F2-F3	13	Azathioprine	12	100	10	193	460	2.38	UD	Azathioprine	150	10	0
3	F2-F3	14	Azathioprine	18	150	10	178	782	4.39	UD	Azathioprine	200	10	0
11	F1-F2	15	Azathioprine	36	75	10	197	996	5.6	UD	Azathioprine	100	10	0
12	F4	16	6-MP	16	50	0	156	1354	8.68	UD	6-MP	75	0	0
13	F0	17	Azathioprine	60	100	5	130	1592	12.25	Shunting	Azathioprine	100	0	0
14	F0	18	Azathioprine	24	100	5	358	672	1.88	Response	Azathioprine	100	5	0
15	F0	19	6-MP	36	50	5	220	3013	13.7	response	6-MP	50	7.5	0
16	F1	20	Azathioprine	48	100	0	714	628	0.84	Toxic dose	Azathioprine	75	0	0
17	F3	21	Azathioprine	12	150	20	269	1574	5.85	Response	Azathioprine	150	10	0
18	F3-F4	22	Azathioprine	60	150	25	277	1175	4.24	Response	Azathioprine	150	25	0
19	F0	23	Azathioprine	3	100	15	137	4344	31.71	Shunting	Azathioprine	100	10	100
20	F3	24	Azathioprine	48	100	5	511	568	1.11	Toxic dose	Azathioprine	50	10	0
21	F3-F4	25	Azathioprine	12	75	12.5	146	726	4.97	UD	Azathioprine	100	12.5	0

6-MP, 6-mercaptopurine; UD, under dosing.

^*^Alternating daily dose.
